# Atherosclerotic Extension of Carotid Arteries: An Insertion in Clinical Practice

**DOI:** 10.1155/2020/3120327

**Published:** 2020-06-23

**Authors:** M. L. Furlanetto, E. F. B. Chagas, Payão SLM

**Affiliations:** Faculdade de Medicina de Marília, Marília, São Paulo, Brazil

## Abstract

**Introduction:**

Atherosclerotic disease is a diffuse disease that is strongly associated with age, risk factors, and variable progression. The anatomical prevalence of atheromas does not always follow, a sequence by sectors, and in many cases are concomitant.

**Objectives:**

This study is aimed at studying atherosclerosis in the arterial territories of the carotid and lower limbs, in order to correlate their extension as a form of primary prevention.

**Methods:**

Participating patients with the main risk factors for atherosclerotic disease were composed of two groups: one with chronic peripheral obstructive arterial disease (PAD) and another without PAD. After performing carotid ultrasound Doppler (USD) of all patients, the occasional prevalence of the disease was evaluated. We performed by statistical tests the correlation between the findings in these patients and the risk factors. Obtaining *n* from 226 patients, in which 116 patients are from the PAD group and 110 patients are from the group without PAD.

**Results:**

Our findings add up to 8.8% for lesions over 50% in patients with PAD, with 6.2% over 70% meeting the few published scientific findings. In this study, the correlation was evaluated between carotid stenosis and PAD, in which we observed a positive association. We observed in the studies that the prevalence of moderate and severe carotid stenosis was similar to patients with coronary artery disease (CAD). There are a number of nonclassical risk factors that we do not evaluate, but even studying the traditional ones, we find that they are less than 27% dependent.

**Conclusion:**

Therefore, our study proposes an improvement in the clinical approach of patients with PAD for both the carotid and coronary territory, not using only 2 factors traditional risk factors, for the extension study and to consider the PAD that has 10% dependence alone, as effect and projection of the carotid atherosclerotic plaque.

## 1. Introduction

The atherosclerotic disease is characterized by chronic immune-inflammatory mechanisms that can promote an acute event, such as plaque destabilization, in arterial light, causing an ischemic event. In the clinical investigation of the presence of atherosclerosis, in patients who have previously diagnosed the pathology (coronary, carotid, or lower limb arteries), we use the term atherosclerotic extension study, for prevention purposes [1].

Currently, stroke is the third leading cause of death in the world, with ischemic events being the main cause for around 87% of the cases. Stroke with hemorrhagic origin represents approximately 17% of the cases, and stroke of ischemic origin, the carotid stenosis, represents around 15% to 20% of the cases [2]. The recurrence risk of stroke in survivors with carotid stenosis is around 4 to 15% in the first year after the stroke and 25% after 5 years [2]. Recent studies have reported an annual risk of stroke of 5% for patients with severe stenosis and 1% for moderate stenosis in asymptomatic patients [3, 4].

The inflammation of an unstable carotid plaque may be the link between CAD and carotid stenosis, as unstable carotid plaque is closely associated with the occurrence of CAD before or after surgical treatment of atherosclerotic disease. Therefore, an unstable carotid plaque may be the phenotype of the representation of the presence of systemic inflammation and often accompanies the presence of CAD [5].

Chronic occlusive peripheral arterial disease (PAD) is the third main cause of death by atherosclerosis, followed by CAD and stroke. PAD is indicative of atherosclerosis in other sectors, with a higher risk of stroke and myocardial infarction. The 5-year mortality rate among patients diagnosed with PAD can reach 33.2%, whereas over 70% of deaths can be attributed to cardiovascular events. The risk of cardiovascular events in patients with PAD and CAD is equivalent [6].

Studies of the extension of carotid lesion in PAD for prevention purposes do not yet have a defined protocol, as in people with CAD. In the PAD2019 guideline [7], we have no such indication for investigation, even though it relates that mortality and disability in patients with PAD is higher, and its damages are not only restricted to the elderly,but also occur in young patients [7].

We know that PAD is a systemic disease and that it represents a high-risk factor for cardiovascular events [8–10], and we have only a few studies addressing this association, with evidences for the prevalence of atherosclerotic disease, which is higher in patients with PAD than in patients without PAD [11–13].

## 2. Patients and Methods

The present work is a cross-sectional, analytical, and observational study, in which the tabulation of the obtained data was used to study the correlation of risk factors, carotid stenosis degrees, PAD, and atherosclerotic disease.

This study was submitted to the Research Ethics Committee of the Marilia Medical School (FAMEMA) and approved (case number: 2.655.740) and carried out at the same institution.

This work is in accordance with the Declaration of Helsinki (1957). Informed consent was duly signed by each participant.

A total of 242 patients were prospectively selected in a sequential manner and later, when convenient, based on gender and age matching, with a variation for every 4 years. The selected patients were over 45 years of age and attended the FAMEMA Vascular Surgery Outpatient Clinic from July 2018 until August 2019.

As inclusion factors, the selected patients presented with absence diagnosis of PAD (presence of all arterial pulses of the lower limbs and absence of lameness of the lower limbs) and presented at least 2 risk factors for atherosclerotic disease (systemic arterial hypertension, type 2 diabetes, smoking or previously smoked, obesity, more than 65 years old, and CAD), as well as other patients diagnosed with PAD.

Exclusion factors included patients already diagnosed with carotid stenosis or carotid-related stroke, or patients with symptoms of transient brain ischemia with syncope, fleeting amaurosis, dizziness, and patients with known history of thrombophilia, vasculitis, or with inherited and acquired connective tissue disorders.

We used the study in the General Vascular Surgery outpatient clinic of the Faculdade de Medicina de Marília (FAMEMA), in which we interviewed all patients who had any vascular disease that was being treated and being followed up. But, in the study, only asymptomatic patients were selected for carotid stenosis. Both for the group without PAD and for the group with PAD:

Patient with any vascular disease, (except PAD) with at least 2 risk factors for atherosclerotic disease, and who never had a stroke or symptoms of carotid origin (Group 1).

Patient diagnosed with PAD, asymptomatic for carotid stenosis, who never had a carotid stroke (Group 2).

We excluded 16 patients using these criteria, achieving a final *N* of 226 patients.

The patients were divided into 2 groups: (1) patients with PAD (*n* = 113) and (2) patients without PAD with at least 2 risk factors for EA (*n* = 113).

We took the patients' clinical histories, reviewed their medical records, and elaborated categorical questions. In addition to the traditional risk factors, we include nontraditional factors such as obesity and abdominal aortic aneurysm (AAA), which presents a chronic inflammatory process in its wall, with thrombus production, and classifies the patient for a high risk of developing atherosclerosis [9, 10].

When in doubt about the diagnosis of type 2 diabetes and dyslipidemia, when collecting this information in specific cases, we requested laboratory tests, such as fasting blood sugar test, glycosylated hemoglobin test, and lipid profile test, which were collected according to the protocol of Marília's Blood Center.

We performed weight measurements in all patients with the same portable scale, as well as height measurements, with the same measure tape, to calculate the body mass index and abdominal circumference measure.

The classification of abdominal circumference, body mass index, dyslipidemia, systemic arterial hypertension, T2DM, and tobacco smoking were defined according to the current international guidelines [7, 10, 14–16].

We performed the carotid DUS of all patients, according to the Brazilian Society of Cardiology [16] protocol, in the moment of evaluation, by the same examiner and using the same device.

We used a handheld DUS device, the *GE logiq E 5417728-100*, plus linear transducer (9 MHz). To measure the carotid intima-media complex (CIMT), we used 5 manual measurements, with a distance of 1 cm prebifurcation. When an atherosclerotic lesion was noted, we associated the analysis of B mode images, as well as color mode and spectral analysis, performing the ratio of systolic peaks, by the NASCET method [17–19].

The NASCET method uses the classification of degrees of stenosis, in intervals, in which they are measured by the ratio of the systolic peak velocity (PSV) of the internal carotid (postinjury) to the PSV of the flow before the injury, in the common carotid artery (if the ratio shows <2 = injury < 50%, if ratio of 2-4 = injury 50-69%, and if reason>4 = injury greater than or equal to 70%). But, we note that this flowmetry study alone is not enough to determine the diagnosis [16–19].

We must always correlate with the B mode and the study of the color mode. Because the tortuous arteries, some hemodynamic states, and the fact that the diagnostic test, being a dependent observer [17–19], does not accurately represent carotid stenosis. In this study, for all patients with an injury of 70% or more, diagnostic arteriography by intra-arterial catheter was performed, and subsequently all were treated with endarterectomy.

We processed the ultrasound results according to the NASCET method [17, 18] although with a modification (mod): within the normal range (without lesions and CIMT < 0.9); CIMT thickness (0.9-1.4); presence of atherosclerosis classified according to lesion intensity: mild atheromatosis (atheromatous plaque > 1.5 mm), mild stenosis (less than 50%), moderate stenosis (between 50 and 69%), severe stenosis (lesion ≥ 70%), and subocclusion/occlusion. Lesions higher than 70% will be added to the findings of subocclusion and occlusion. In occlusions, the patient does not need to perform invasive treatment, but the aim of this study is to evaluate the prevalence of lesions, even if they have progressed to over 70%.

In the tabulation of each patient, we consider the largest lesion in 1 artery to account for the stratification of the lesions and to prevent the patient who has several changes from being counted more than once in the statistics.

For the diagnosis of PAD, besides the anamnesis, the study of medical records, in which we observed previous PAD treatments (endovascular procedures, revascularization surgeries, and amputations) for a palpation of the arterial pulses, we performed the ankle-brachial index calculation with portable Doppler, and when necessary, we used the lower limb arterial DUS [7].

The clinical classification used for PAD was Rutherford, which consists of the following: Rutherford 0: asymptomatic, Rutherford 1: mild lameness (>400 meters), Rutherford 2: moderate lameness (200-400 meters), Rutherford 3: severe lameness (<200 meters), Rutherford 4: resting pain, Rutherford 5: minimal trophic injury, and Rutherford 6: major trophic injury [9].

We chose not to map or compare between the groups, the medications, their time of use, and the time of diagnosis of the base lesions, as it is a reference service for vascular surgery of several medical centers in the system of public health in the region, in which we do not know the real consistency of these basic treatments.

It was not possible to analyze the medications and the time of use, because in this outpatient clinic, we mostly serve patients with very low income and low social and even cognitive conditions, in whom they do not know how to inform the correct names of the medicines and the form of administration. They suffer from the oscillation of gaining or not from SUS, the medications for continuous use, and go months without the proper use of these medications. So, we chose not to consider the effect of the medications in this study because it presents this VIES of patients' adherence to basic medications.

The basic drugs used and given to these patients in systemic arterial hypertension are losartan, hydrochlorothiazide, and propanolol; in diabetes mellitus, metformin, glibenclamide, and NPH and regular insulin; and in dyslipidemia, only simvastatin and ciprofibrate.

### 2.1. Statistical Analysis

Quantitative data was described through mean and standard deviation (SD), and a comparison of independent media was performed by the nonparametric Mann–Whitney test. Qualitative variables are described by absolute (*N*) and relative (%) frequency distribution and their association analyzed by the Chi-square test. The selected linear regression model was used to analyze the effect of (independent) covariates on a dependent variable. The significance level adopted was 5% (*p* ≤ 0.05), and the results were analyzed using the SPSS version 19.0 software. The sample size (*n*) was determined considering an average effect size (0.30) [12], a type I (*α*) error margin of 5%, and a study power of 80%, indicating the minimum need of 108 sampled elements per group. The peak systolic velocity (PSV) was described by the median and quartile distribution in the box plot chart. The comparison between the degrees of stenosis for PSV was performed by the Kruskal-Wallis nonparametric test and the Holm-Sidak post hoc test.

## 3. Results

**Figure 1:**
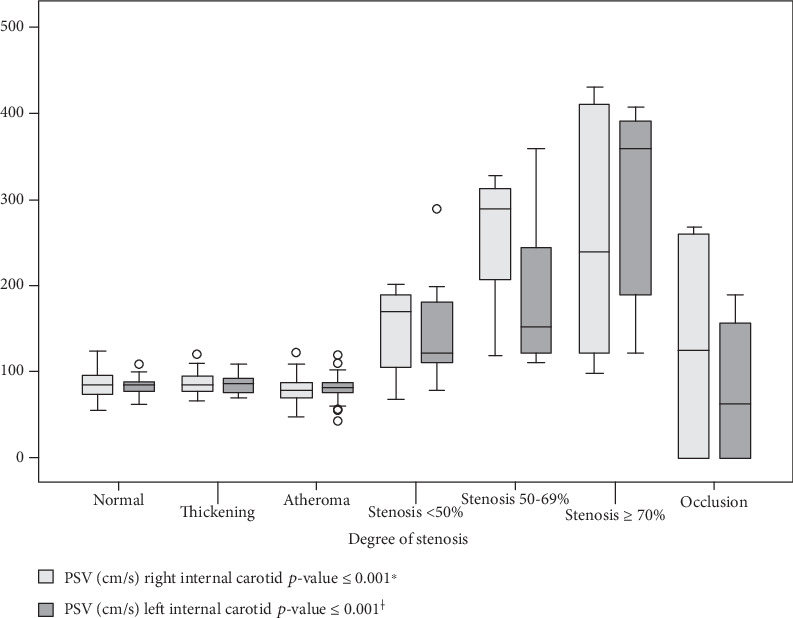
Box plot of the peak systolic velocity (cm/s) (*y*-axis) by the degree of stenosis. Significant differences for PSV (cm/s) between degrees of stenosis by the Kruskal-Wallis nonparametric test for the right internal carotid (^∗^) and for left internal carotid (^†^).

There were no significant differences between the degrees of normal stenosis, thickening, atheroma, and occlusion for peak systolic velocity in the right internal carotid (ICR) and left internal carotid (LIC). In RIC, stenosis < 50% had PSV values higher than normal, thickening, and atheroma, but less than 50-69% and stenosis ≥ 70%. The degrees of stenosis of 50-69% and ≥70% did not show differences for PSV in RIC; however, they were higher than the other degrees. In LIC, the PSV values of the degree of stenosis < 50% were higher than normal, thickening, atheroma, and occlusion and lower than the degrees of stenosis of 50-69% and ≥70%. The PSV values in the LIC of the degrees of stenosis 50-69% and ≥70% were higher than the other degrees; however, the PSV in the LIC of the degree of stenosis ≥ 70% was higher than the degree of stenosis of 50-69% (Figure 1).

### 3.1. Clinical and Demographic Characteristics

The youngest patient was 47 years old and the oldest was 89 years old, with the average age being 65.5 ± 9.3. The sample distribution of gender and age variables was homogeneous due to the pairing, in which each contained 113 individuals, being 58 men and 55 women in each group, and a total of 226 people.

The groups were matched for gender and age. During the initial phase the sample was randomized by the sequence of visits to the FAMEMA Vascular Surgery outpatient clinic, and as the sample began consolidating, the proper pairing was considered. From the group with PAD, 59 patients (52.3%) presented a mild and/or moderate disease, with intermittent claudication symptoms; 30 patients (26.5%) were considered severe and classified as critical ischemia, and 24 (21.2%) patients were asymptomatic. Only 10 (8.8%) patients underwent previous revascularization with conventional bypass surgery, 24 patients (21.2%) underwent previous angioplasty, and 20 patients (17.7%) had some type of amputation.

All patients with the absence of neurological symptoms or the presence of already known carotid stenosis were included.

The prevalence of stroke in the PAD group was double that of the other group at the time of clinical evaluation.

In the results, the stroke findings are all confirmed to be noncarotid; otherwise, they would be excluded from the study.

### 3.2. Variables

In our results, we did not show a statistically significant increase for age and gender, systemic arterial hypertension, type 2 diabetes, preexisting conditions, obesity, CAD, and abdominal aortic aneurysm in relation to PAD, as shown in Table 1. Although gender, obesity, and AAA are not considered traditional risk factors, like the other ones [20].

Tobacco smoking is a very clear risk factor in the development and rapid progression of atherosclerosis. In our findings, a statistically significant increase was evidenced for the PAD group, with a prevalence of 84.1% (Table 2).

The prevalence of hypertension in the PAD group was 81.4%, type 2 diabetes was 47.8%, dyslipidemia 53.1%, obesity 31%, and AAA 8.0%, but none of these factors showed a statistically significant difference (Table 2).

In Table 2, the PAD group showed significantly higher values compared to the group without PAD when it comes to risk factors and carotid stenosis degree.

When we look at obesity (abdominal circumference + body mass index), we show a paradoxical effect and a protective effect, as it has a positive correlation for the group without PAD and a negative correlation for the group with PAD.

In Table 3, we tabulate the results of the associations of the sonographic results according to NASCET in relation to the group with PAD and without PAD. We found a positive correlation in our results for all possible changes and degrees of stenosis, in the carotid DUS, for the group of patients with PAD. The examination with normal results (no changes) has a statistically significant value for the group without PAD.

We performed linear regression analysis to study the dependencies of variables with PAD as per Table 4. In the analysis of the general model, it was observed that the presence of PAD, tobacco smoking, type 2 diabetes, AAA, and the male gender, as well as the increase in age, contribute significantly to the increase of the degree of stenosis. These variables together account for 26.5% (*R*^2^) of the variation in the degree of stenosis. However, PAD alone had a significant effect on the degree of stenosis, accounting for 10.4% (R2) for the variation in the degree of stenosis.

With the insertion of the covariates age, sex, tobacco smoking, type 2 diabetes, and AAA, it was found that these together account for 22.6% (*R*^2^) of the variation of the degree of stenosis.

When analyzing the effect of the Rutherford scale on the degree of stenosis in patients with PAD, no significant effect was observed.

However, by inserting the covariates: age, sex, tobacco smoking, type 2 diabetes, stroke, and AAA, the regression model was significant to predict the variations in the degrees of stenosis by 17% (*R*^2^). But when analyzing the independent variables, it was observed that only the covariates gender, type 2 diabetes, and stroke were actually significant.

## 4. Discussion

It is clear that atherosclerosis is a chronic multifactorial disease, which still includes other factors not addressed in this study. When we categorically observe the prevalence and associations between them with PAD, risk factors do not seem to have such a relevant force when we consider the classic factors in this study. For only tobacco smoking had a statistically significant value, and in regression models, working with several associated factors, are dependent on less than 27% of stenosis carotid.

In the results of this study, we observed that the PAD group presented a statistically significant difference for all degrees of stenosis and predictive changes of stenosis carotid in this group, the correlation was positive (Table 3).

The results of the present study add up to 8.8% for lesions above 50% in patients with PAD, and 6.2% over 70%, which, to date, meet the few published findings, but with lower values when compared to published studies ranging from 5 to 24% in patients with PAD [11–13]. In the Present Review of Asymptomatic Carotid Stenosis (2020), the authors found 13.4% prevalence of moderate stenosis and 21.8% for severe stenosis [21]. While there is, in the general population, a low prevalence of asymptomatic carotid stenosis, with 4.2% of the population with moderate stenosis (>50%) and 1.7% with severe stenosis (>70%) [22]. Because our PAD group had all the different possible stages of the disease, and most studies of both cardiology and vascular surgery had studied critically ill patients, whether severe coronary artery disease or already revascularized lower limb ischemia, we believe this strengthens Hamada et al.'s hypothesis [5], in which an unstable plaque can induce systemic inflammation and decompensate other plaques in other sectors, even months after instability.

Based on the obtained results, and the few studies that show the prevalence of asymptomatic carotid stenosis (with risk of complication: stenosis > 50%) in patients with PAD, and considering that cardiology has already well established the study of the extension of atherosclerosis to carotid territory in coronary artery disease patients, with a close prevalence rate of asymptomatic carotid stenosis, with stenosis > 50% for CAD, in relation to PAD, our data corroborates the consideration of the extension study for PAD for prophylaxis, once these patients were critically ill with several associated chronic comorbidities, and the PAD alone had a significant effect on the degree of stenosis, accounting for 10.4% dependence on carotid injury.

This study focused on observing the prevalences that are close in the three arterial sectors when we researched their prevalences. At the intersection of these arterial sectors, our findings for the PAD group were 22.1% for prevalence of CAD, even though not statistically significant, the prevalence was similar when comparing PAD x CAD and CAD x carotid stenosis [5, 11, 23–25]. The group that held patients with PAD had 25% of CAD and 15% more strokes than the group without PAD. In our study, this ratio was double.

We categorically evaluated and could have higher prevalences for both CAD and carotid stenosis asymptomatic if, respectively, we evaluated the coronary arteries using imaging exams, and also qualitatively evaluated the carotid atherosclerotic plaque, with the aid of software, which could increase the diagnosis of plaque prone to complications. Nevertheless, with a high risk of morbidity for cardiovascular events, patients with PAD are usually inadequately evaluated by the vascular surgeon when compared with patients with CAD [23]. Therefore, our study proposes an improvement in the clinical approach of patients with PAD for both the carotid and coronary territory.

## 5. Conclusion

In this study, the correlation was assessed between carotid stenosis and PAD, in which a positive association was observed. There was statistical significance in the correlation of PAD and all degrees of carotid stenosis. The two groups had a prevalence of very close risk factors, and the PAD group had twice as many patients with moderate and severe stenosis, compared to the group with only two risk factors. It was found that the prevalence of moderate and severe carotid stenosis in patients with PAD was similar to patients with CAD. There are numerous nonclassical risk factors that have not been evaluated, but even studying only the traditional ones, it was found that these depend on less than 27%. The finding of the obesity paradox should be better studied in order to have better evidence for such a hypothesis.

So if we look only at the risk factors, the chance is high of not diagnosing a possible carotid lesion with a risk of stroke, and if we add the PAD with an important factor, which increases the prevalence of carotid stenosis, we suggest the extension study carotid atherosclerotic disease for patients with PAD. Even so, with a high probability of morbidity and mortality for stroke, patients with PAD are generally inadequately assessed by the vascular surgeon, compared to patients with CAD. Finally, the present study proposes an improvement in the clinical approach of patients with PAD, both for the carotid and coronary territories.

## Figures and Tables

**Table 1:** Analysis of the association between the presence of PAD and the variables (risk factors/diseases).

	Groups		*p* value
	Not PAD	PAD	
Tobacco smoking			
*N*	74	95	0.001^∗^
%	65.5%	84.1%	
Dyslipidemia			
*N*	49	60	0.144
%	43.4%	53.1%	
Systemic arterial hypertension			
*N*	88	92	0.510
%	77.9%	81.4%	
T2DM			
*N*	48	54	0.424
%	42.5%	47.8%	
Stroke			
*N*	8	14	0.179
%	7.1%	12.4%	
CAD			
*N*	15	25	0.082
%	13.3%	22.1%	
Obesity			
*N*	52	35	0.020^∗^
%	46.0%	31.0%	
AAA			
*N*	18	9	0.066
%	15.9%	8.0%	

Note:^∗^*p* value ≤ 0.05 indicates significant association by the Chi-square test. ^†^*p* value ≤ 0.05 indicares significant association by Fisher's exact test. AAA: abdominal aortic aneurysm.

**Table 2:** Comparison between patients with and without PAD in relation to carotid stenosis, age, number of risk factors, abdominal circumference, and body mass index.

	Not PAD (*n* = 113)		PAD (*n* = 113)		*p* value
	Mean	SD	Mean	SD	
Age (years)	65.5	9.3	65.6	9.0	0.931
Body mass index	30.5	7.4	27.0	5.8	<0.001^∗^
Abdominal circumference	103.9	13.8	97.5	16.4	0.001^∗^
Number of risk factors	3.4	1.2	3.8	1.3	0.010^∗^
Carotid stenosis degree	1.7	1.2	2.5	1.1	<0.001^∗^

Note:^∗^significant difference between groups by the nonparametric Mann–Whitney test. Carotid stenosis degree represents variable on ordinal scale (0 = normal, 1 = thickening, 2 = atheroma, 3 = stenosis < 50%, 4 = stenosis 50 to 69%, 5 = stenosis ≥ 70%, and 6 = occlusion).

**Table 3:** Association between PAD and carotid stenosis degree.

Degree	COPAD group		*p* value
	Not COPAD	COPAD	
Normal			<0.001^∗^
*N*	25	4	
%	22.1%	3.5%	
CIMT thickening			
*N*	19	7	
%	16.8%	6.2%	
Atheroma			
*N*	44	58	
%	38.9%	51.3%	
Stenosis < 50%			
*N*	20	27	
%	17.7%	23.9%	
Stenosis 50-69%			
*N*	2	10	
%	1.8%	8.8%	
Stenosis ≥ 70%			
*N*	2	4	
%	1.8%	3.5%	
Occlusion			
*N*	1	3	
%	0.9%	2.7%	

^∗^ *p* value ≤ 0.05 indicates significant association by the Chi-square test.

**Table 4:** Linear regression analysis for the effect of independent variables on the degree of obstruction for the total sample (general model) and for patients with PAD (PAD model).

Variables		Regression coefficient				Model	
		*B*	95.0% CI for		*p* value	*p* value	*R* ^2^
Dependents	Independent		LI	LS			
Degree (general model)	(constant)	1.69	1.47	1.911	<0.001^∗^	<0.001^∗^	0.104
	PAD	0.805	0.494	1.117	<0.001^∗^		

Degree (general model)	(constant)	-1.237	-2.369	-0.104	0.032^∗^	<0.001^∗^	0.265
	PAD	0.743	0.447	1.04	<0.001^∗^		
	Age (years)	0.033	0.017	0.049	<0.001^∗^		
	Sex	0.428	0.134	0.721	0.004^∗^		
	Tobacco smoking	0.376	0.021	0.73	0.038^∗^		
	T2DM	0.574	0.281	0.866	<0.001^∗^		
	AAA	0.524	0.07	0.978	0.023^∗^		

Degree (general model)	(constant)	1.855	1.65	2.061	<0.001^∗^	<0.001^∗^	0.056
	Rutherford	0.168	0.077	0.26	<0.001^∗^		

Degree (general model)	(constant)	-1.168	-2.33	-0.006	0.048^∗^	<0.001^∗^	0.226
	Rutherford	0.152	0.066	0.239	0.001^∗^		
	Age (years)	0.033	0.016	0.049	<0.001^∗^		
	Sex	0.434	0.132	0.736	0.005^∗^		
	Tobacco smoking	0.483	0.125	0.842	0.008^∗^		
	T2DM	0.571	0.271	0.872	<0.001^∗^		
	AAA	0.486	0.019	0.953	0.041^∗^		

Degree (PAD model)	(constant)	2.637	2.172	3.103	<0.001^∗^	0.496	0.004
	Rutherford	-0.05	-0.197	0.096	0.498		

Degree (PAD model)	(constant)	0.393	-1.339	2.126	0.654	0.005^∗^	0.170
	Rutherford	-0.052	-0.193	0.089	0.463		
	Age (years)	0.022	-0.001	0.045	0.062		
	Sex	0.446	0.02	0.873	0.040^∗^		
	Tobacco smoking	0.231	-0.372	0.834	0.449		
	T2DM	0.531	0.11	0.952	0.014^∗^		
	Stroke	0.781	0.17	1.392	0.013^∗^		
	AAA	0.392	-0.385	1.17	0.319		

*B*: regression coefficient; 95% CI: 95% confidence interval for B; LI: limit inferior; LS: limit superior. ^∗^*p* value ≤ 0.05 significant effect of the independent variable by the Wald statistic. ^†^*p* value ≤ 0.05 indicates that the model is significant for predicting the dependent variable. Degree is as follows: 0: normal; 1: C-IMC thickening; 2: atheroma; 3: stenosis < 50%; 4: stenosis 50-69%; 5: stenosis ≥ 70%; and 6 = occluded). Rutherford is as follows: 0: no COPAD; 1: Rutherford 0 (asymptomatic); 2: Rutherford 1; 3: Rutherford 2; 4: Rutherford 3; 5: Rutherford 4; and 7 = Rutherford 6. Gender (0 = female; 1 = male). Tobacco smoking (0 = nonsmoker; 1 = smoker). T2DM. CVA and AAA (0 = absent, 1 = present).

## Data Availability

The (DATA TYPE) data used to support the findings of this study are included within the article.
